# Evidence for *ACTN3* as a genetic modifier of Duchenne muscular dystrophy

**DOI:** 10.1038/ncomms14143

**Published:** 2017-01-31

**Authors:** Marshall W. Hogarth, Peter J. Houweling, Kristen C. Thomas, Heather Gordish-Dressman, Luca Bello, Cooperative International Neuromuscular Research Group (CINRG), V. Vishwanathan, S. Chidambaranathan, W. Douglas Biggar, Laura C. McAdam, Jean K. Mah, Mar Tulinius, Avital Cnaan, Lauren P. Morgenroth, Robert Leshner, Carolina Tesi-Rocha, Mathula Thangarajh, Tina Duong, Andrew Kornberg, Monique Ryan, Yoram Nevo, Alberto Dubrovsky, Paula R. Clemens, Hoda Abdel-Hamid, Anne M. Connolly, Alan Pestronk, Jean Teasley, Tulio E. Bertorini, Richard Webster, Hanna Kolski, Nancy Kuntz, Sherilyn Driscoll, John B. Bodensteiner, Jose Carlo, Ksenija Gorni, Timothy Lotze, John W. Day, Peter Karachunski, Erik K. Henricson, Richard T. Abresch, Craig M. McDonald, Elena Pegoraro, Eric P. Hoffman, Stewart I. Head, Kathryn N. North

**Affiliations:** 1 Institute for Neuroscience and Muscle Research, The Children's Hospital Westmead, New South Wales 2145, Australia; 2 Discipline of Paediatrics and Child Health, Faculty of Medicine, University of Sydney, New South Wales 2006, Australia; 3 School of Medical Sciences, University of New South Wales, New South Wales 2052, Australia; 4 Murdoch Childrens Research Institute, Melbourne, Victoria 3052, Australia; 5 Department of Paediatrics, Faculty of Medicine, Dentistry and Health Sciences, University of Melbourne, Melbourne, Victoria 3010, Australia; 6 Research Centre for Genetic Medicine, Children's National Medical Centre, Washington DC 20010, USA; 7 Department of Neurosciences, University of Padova, Padova 35122, Italy; 8 Sundaram Medical Foundation and Apollo Children's Hospital, Chennai, India; 9 Holland Bloorview Kids Rehab Hospital, Toronto, Ontario, Canada; 10 Alberta Children's Hospital, Calgary, Alberta, Canada; 11 Queen Silvia Children's Hospital, Göteborg, Sweden; 12 Children's National Medical Center, Washington DC, USA; 13 Royal Children's Hospital, Melbourne, , Victoria, Australia; 14 Hadassah Hebrew University Hospital, Jerusalem, Israel; 15 Instituto de Neurosciencias Fundacion Favaloro, Buenos Aires, Argentina; 16 University of Pittsburgh and Department of Veterans Affairs, Pittsburgh, Pennsylvania, USA; 17 Washington University in St Louis, St Louis, Missouri, USA; 18 Children's Hospital of Virginia, Richmond, Virginia, USA; 19 University of Tennessee, Memphis, Tennessee, USA; 20 Children's Hospital at Westmead, Sydney, New South Wales, Australia; 21 University of Alberta, Edmonton, Alberta, Canada; 22 Mayo Clinic, Rochester, Minnesota, USA; 23 University of Puerto Rico, San Juan, Puerto, Rico; 24 University of Pavia and Niguarda Ca' Granda Hospital, Milan, Italy; 25 Texas Children's Hospital, Houston, Texas, USA; 26 University of Minnesota, Minneapolis, Minnesota, USA; 27 University of California, Davis, Sacramento, California, USA

## Abstract

Duchenne muscular dystrophy (DMD) is characterized by muscle degeneration and progressive weakness. There is considerable inter-patient variability in disease onset and progression, which can confound the results of clinical trials. Here we show that a common null polymorphism (R577X) in *ACTN3* results in significantly reduced muscle strength and a longer 10 m walk test time in young, ambulant patients with DMD; both of which are primary outcome measures in clinical trials. We have developed a double knockout mouse model, which also shows reduced muscle strength, but is protected from stretch-induced eccentric damage with age. This suggests that α-actinin-3 deficiency reduces muscle performance at baseline, but ameliorates the progression of dystrophic pathology. Mechanistically, we show that α-actinin-3 deficiency triggers an increase in oxidative muscle metabolism through activation of calcineurin, which likely confers the protective effect. Our studies suggest that *ACTN3* R577X genotype is a modifier of clinical phenotype in DMD patients.

## Abstract (web summary)

Duchenne muscular dystrophy is a disease caused by a single gene, characterized by progressive muscle weakness, but is variable between patients partly due to interactions of other genes. Here, the authors show that a common *ACTN3* polymorphism can modify the clinical phenotype.

Duchenne muscular dystrophy (DMD) is an X-linked genetic disorder characterized by progressive muscle degeneration and weakness. DMD is caused by mutations in the *DMD* gene, resulting in loss of dystrophin protein at the muscle membrane^1^. To date, the only proven palliative therapy for DMD is chronic corticosteroid (that is, prednisone or deflazacort) treatment^2^,^3^, the effects of which can be highly variable between patients. There is also considerable inter-patient variability in disease onset and progression^4^. This presents a significant challenge to clinicians in counselling patients and threatens to mask the benefits of novel treatments, and confound the results of clinical trials.

It has long been hypothesized that genetic variations at loci independent of dystrophin (that is, genetic modifiers) are responsible for unexplained variability in the progression of muscle weakness and response to therapies in patients with DMD. The first disease modifying gene to be identified in DMD patients, osteopontin (*SPP1*), was reported in 2011. A single nucleotide polymorphism (SNP) in *SPP1*, resulting in reduced gene expression, was associated with greater muscle weakness and earlier loss of ambulation (LoA)^5^. The mechanism underlying this effect on DMD phenotype has not yet been determined, but *SPP1* expression is known to affect muscle regeneration^6^ and is upregulated in DMD patients^7^. Importantly, when stratifying cohorts based on *SPP1* genotype, the number of patients required to detect a significant difference in a clinical trial setting is reduced by roughly 75%. More recently, *LTBP4* was found to influence the age at which patients with DMD became non-ambulant via modulation of the TGF-β signalling pathway^8^,^9^. The modifier effect on LoA has since been replicated for both of these genes in a large patient cohort, but only when the cohort was stratified for both ethnicity and corticosteroid treatment, highlighting the difficulty associated with identifying genetic modifiers.

Interestingly, both *SPP1* and *LTBP4* map to the TGF-β pathway, a key driver of failed regeneration and the development of fibrosis in DMD (ref. 10), highlighting the potential for genetic modifiers to reveal molecular pathways which drive the dystrophic pathology. The ongoing identification of modifier genes is therefore of considerable importance as they contribute to our understanding of the pathological mechanisms that underpin DMD, and will have a major influence on future clinical trial design.

In skeletal muscle, the sarcomeric α-actinins are the major components of the Z-line where they bind and crosslink the actin thin filaments. α-Actinin-2 is expressed in all muscle fibres, but the highly homologous α-actinin-3 isoform is expressed only in fast, glycolytic fibre types. Importantly, there is a common null polymorphism (R577X, rs1815739) in the gene coding for α-actinin-3 (*ACTN3*), resulting in complete absence of α-actinin-3 in homozygous individuals (*ACTN3* 577XX, ∼16% of the global population and ∼18% of Caucasians)^11^. α-Actinin-3 deficiency is detrimental to sprint and power performance in both elite athletes^12^,^13^ and the general population^14^,^15^,^16^. Conversely, α-actinin-3 deficiency is associated with increased endurance performance in some athlete cohorts^13^,^17^, and improved response to resistance training^14^. *Actn3* knockout (KO) mice mimic the phenotype seen in α-actinin-3 deficient humans; *Actn3* KO mice have reduced muscle mass and strength^18^, but display increased endurance capacity, fatigue resistance and response to training^19^,^20^.

*Actn3* KO mice do not exhibit a change in fibre type distribution at baseline, but the fast 2B fibres, which would usually express α-actinin-3, display a shift towards the properties classically associated with slower fibre types including; reduced fibre diameter^18^, increased oxidative metabolism^18^,^21^,^22^, slowed relaxation from contraction^23^ and an increased recovery from fatigue^19^. The mechanism responsible for this shift in fast-to-slow properties is likely because of a combination of metabolic and signalling changes in the absence of α-actinin-3. Specifically, we have identified increases in glycogen storage (because of a reduction in glycogen phosphorylase activity)^22^ along with increased calcineurin activity in *Actn3* KO muscle^20^, which together promote greater oxidative metabolism and a fast-to-slow fibre type conversion in skeletal muscle.

It is clear that *ACTN3* genotype significantly influences muscle function in both elite athletes and healthy individuals and hence may also be capable of modifying muscle function in DMD patients. Slower, more oxidative fibres are relatively spared from degeneration in DMD (ref. 24). In addition, calcineurin overexpression in *mdx* mice has been demonstrated to ameliorate the dystrophic phenotype^25^,^26^. Taken together these data led us to hypothesize that *ACTN3* is capable of acting as a genetic modifier of DMD, and that the shift towards slower muscle metabolic properties associated with α-actinin-3 deficiency may have a protective effect against the pathogenesis of DMD. Here we show that the common null polymorphism (R577X) in *ACTN3* results in significantly reduced muscle strength and a longer 10 m walk test time in young, ambulant patients with DMD. Using an *Actn3* KO *mdx* (double KO) mouse model, we show a similar reduction in muscle strength, but protection from stretch-induced eccentric damage with age. Mechanistically, α-actinin-3 deficiency triggers an increase in oxidative muscle metabolism through activation of calcineurin, which likely confers the protective effect. This work therefore suggests that α-actinin-3 deficiency reduces muscle strength performance, but ameliorates the progression of dystrophic pathology.

## Results

### *ACTN3* genotype modifies strength in ambulant DMD patients

To assess muscle function in DMD, we analysed baseline data on a cohort of DMD patients (*n*=272) enroled in the Cooperative International Neuromuscular Research Group (CINRG) DMD natural history study (DNHS). We stratified this cohort to include steroid treated, ambulant, (young) 6–10 year old (*n*=61) and analysed quantitative muscle testing (QMT) and timed functional testing (10 m walk velocity); this approach is considered to provide the most sensitive and reliable phenotype to assess genetic modifiers^27^.

At baseline, *ACTN3* genotype had a significant effect on both isolated muscle strength (grip QMT, knee and elbow extensor QMTs) and overall muscle function (10 m walk test). In all cases, the *ACTN3* 577X allele was associated with poorer muscle performance; *ACTN3* 577RX patients display significantly less strength across the QMTs and took significantly longer to complete the 10 m walk test compared with patients with *ACTN3* 577RR genotype. Similarly, patients with the *ACTN3* 577XX genotype show a trend for reduced muscle power output across all outcome measures in the cohort (Table 1).

### Young dKO mice display reduced strength

**Figure 1:**
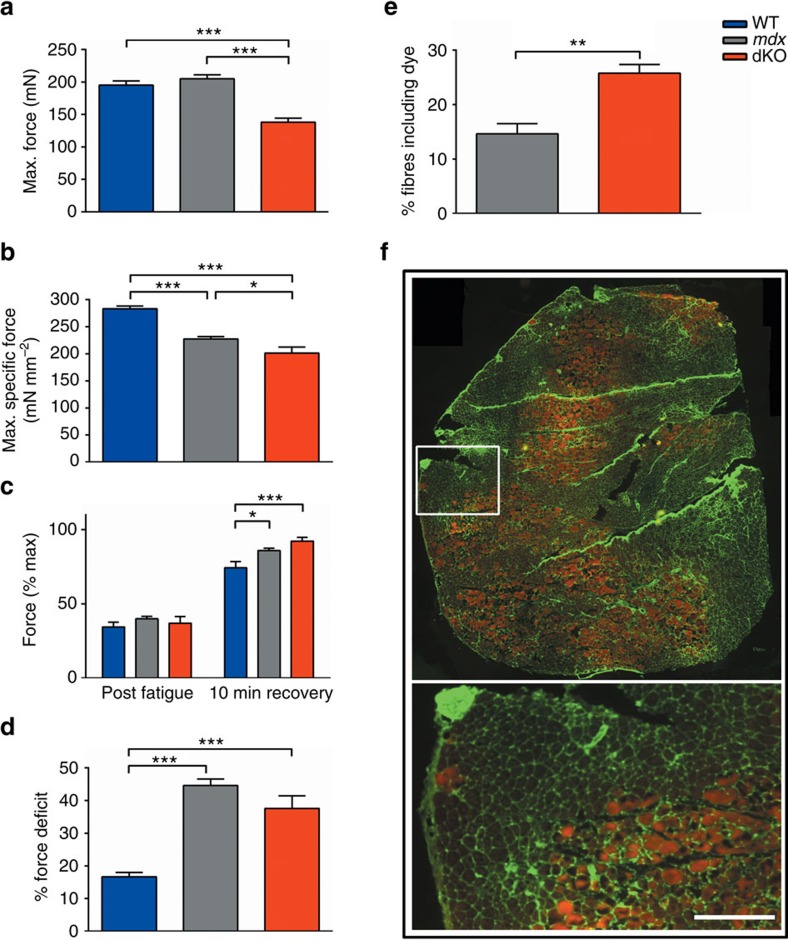
Baseline muscle physiology from 8 week old mice. (**a**) dKO muscles produce ∼33% less force than *mdx* controls (*mdx* - 204.9 mN, dKO - 138.3 mN, *P*<0.001). (**b**) When corrected for cross-sectional area, dKO muscles produce significantly less maximum specific force than *mdx*. (**c**) Both *mdx* and dKO show significantly greater recovery from fatigue than WT, but no difference was observed between *mdx* and dKO. (**d**) dKO muscles suffer slightly less damage than *mdx* controls following a series of 6 20% eccentric contractions (*mdx* 45% drop in force following eccentric contractions, dKO 38%, *P*=0.08). A subset of the WT EDL muscle physiology data was previously published in Hogarth *et al*.^43^ (**e**) A higher percentage of gastrocnemius muscle fibres are EBD-positive following a downhill run in dKO mice than *mdx*. (**f**) Representative cross-section from a dKO gastrocnemius following downhill run. EBD positive fibres are shown in red and wheat germ agglutinin (Green) was used to delineate the fibre border. Scale bar indicates 500 μm. Data shown as mean±s.e.m., One-way ANOVA, *P<0.05, **P<0.01, ***P<0.001. WT *n*=11, *mdx n*=16, dKO *n*=11.

To explore the *ACTN3* genotype effect observed in DMD patients, we crossed *Actn3* KO mice^18^ with the *mdx* mouse model of DMD, to create a double knockout model (dKO) lacking both α-actinin-3 and dystrophin. In agreement with our observations in ambulant DMD patients, muscles from young dKO mice (aged 8 weeks) display significantly reduced force producing capacity compared with *mdx* controls (Fig. 1a), which is maintained when correcting for the reduced size of the dKO muscles (Fig. 1b). Interestingly, both *mdx* and dKO muscles showed increased recovery from fatigue compared with WT (Fig. 1c), but we saw no difference between *mdx* and dKO. We saw no difference in the hill coefficient or twitch time to peak between *mdx* and dKO, but did see an increased half-relax time in dKO compared with *mdx*, indicating slowed relaxation following contraction (Table 2; Supplementary Fig. 1A–C). Dystrophic muscle is known to be vulnerable to stretch-induced damage^28^, and both *mdx* and dKO muscles are injured following a series of 20% eccentric stretches (Fig. 1d). We examined this phenotype further by inducing eccentric contractions *in vivo* via a downhill running protocol, using Evans blue dye (EBD) to mark injured fibres^29^. We saw a significantly increased number of EBD-positive fibres in dKO muscles (Fig. 1e), although this may be a product of the increased endurance capacity associated with α-actinin-3 deficiency^20^. A representative image of skeletal muscle following downhill run shown in Fig. 1f.

### α-Actinin-3 deficiency ameliorates *mdx* disease progression

**Figure 2:**
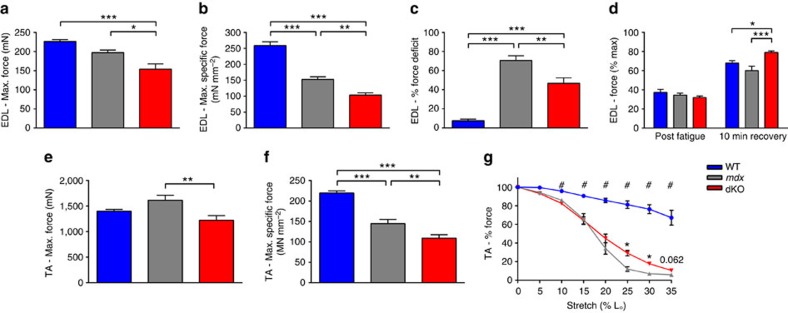
Muscle physiology from 12 month old mice. (**a**–**d**) *Ex vivo* EDL muscle function. (**a**) dKO muscles produce ∼27% less force than *mdx* controls (*mdx* – 171.0 mN, dKO – 126.2 mN, One-way ANOVA, *P*<0.001). (**b**) When corrected for cross-sectional area, dKO muscles produce significantly less maximum specific force than *mdx*. (**c**) dKO muscles suffer less damage than *mdx* controls following a series of three 15% eccentric contractions (*mdx* - 71% drop in force following eccentric contractions, dKO - 47%, One-way ANOVA, *P*<0.01). (**d**) dKO show significantly greater recovery from fatigue compared with WT, and *mdx*. (**e**,**f**) *In situ* TA muscle function replicates the phenotype seen in the EDL. (**e**) dKO muscles produce ∼25% less force than *mdx* controls (*mdx* - 1613 mN, dKO-1221 mN, One-way ANOVA, *P*<0.01), (**f**) After correction for cross-sectional area, dKO show reduced specific force compared with *mdx*. Both are significantly reduced compared with WT. (**g**) Both *mdx* and dKO muscles are significantly injured compared with WT by eccentric contractions of increasing strain (Multiple *T*-test, FDR 1% ^#^*P* <0.01), but dKO muscles suffer less damage than *mdx* between 25-30% L_O_ (0–35%; *mdx* – 80% drop in force following eccentric contractions, dKO 65%, Multiple *T*-test, FDR 1%, *P*<0.05). Data shown as mean±s.e.m., One-way ANOVA, *P<0.05, **P<0.01, ***P<0.001, WT *n*=5, *mdx n*=6, dKO *n*=6.

To determine the influence of α-actinin-3 deficiency on the progression of the dystrophic pathology, we analysed muscle from aged (12 month) mice. Similar to our findings at baseline (8 week old mice), ex vivo EDL muscles of dKO mice produced less force than *mdx* (Fig. 2a), even when corrected for cross sectional area (Fig. 2b). The half-twitch relaxation time was also increased in dKO (Table 3). Importantly, aged dKO muscles demonstrated a significant reduction in force deficit following eccentric contraction compared with *mdx* controls (Fig. 2c). Aged dKO muscles also show considerably greater recovery from fatigue than *mdx* (Fig. 2d). Given that there was no difference in either parameter at 8 weeks, these findings suggest that α-actinin-3 deficiency protects against susceptibility to muscle damage with age. We sought to replicate these findings in the TA, using an *in situ* protocol, which arguably mimics muscle physiology better than the *ex vivo* EDL technique, and again saw reduced absolute force (Fig. 2e), maximum specific force (Fig. 2f), and decreased susceptibility to eccentric damage (Fig. 2g) in dKO compared with *mdx*.

**Figure 3:**
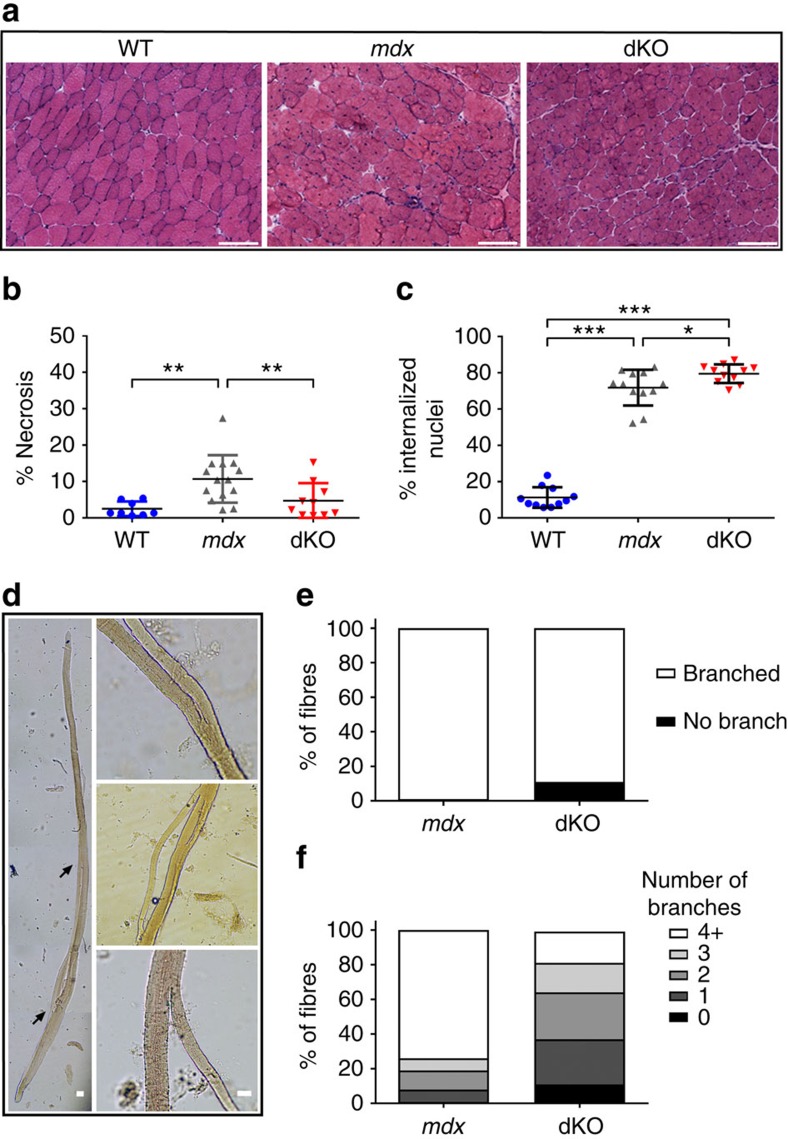
Muscle pathology from aged *mdx* and dKO mice. (**a**) Hematoxylin and eosin (H&E) stained TA sections from WT, *mdx* and dKO mice. Scale bar indicates 100 μm. (**b**) dKO muscles showed a significant reduction in total necrotic area compared with *mdx* (*mdx* – 10.7% versus dKO – 4.7%). (**c**) Both *mdx* and dKO show an increase in centrally nucleated fibres compared with WT, and dKO show a further increase compared with *mdx* (dKO – 79% versus *mdx* 71%, *P*<0.05). Data shown as mean±s.e.m., One-way ANOVA, *P<0.05, **P<0.01, ***P<0.001. WT *n*=11, *mdx n*=12, dKO *n*=11. (**d**) Individual fibres were isolated from 12 month old EDLs to assess fibre branching. The left hand panel shows an entire intact fibre from *mdx* with a branch which originates from the main body of the fibre which re-joins at the mid-belly of the fibre (indicated by arrows). The right hand panels show higher magnification examples of fibre branching. Scale bar indicates 100 μm. (**e**) Almost all fibres isolated from *mdx* (99%) and dKO (89%) are branched. (**f**) However, *mdx* show a higher number of branches per fibre than dKO; 74% of *mdx* fibres have 4 or more branches compared with 18% of dKO. Fibre counts are pooled (*n*=3 mice per genotype) with ∼600 fibres analysed per genotype.

To understand the phenotypes described above, we studied the TA histopathology of *mdx* and dKO mice (Fig. 3a). We observed a significant reduction in necrosis (*mdx*=10.7%, dKO=4.7%, Fig. 3b) and an increase (∼11%) in centrally nucleated fibres in dKO muscle compared with *mdx* (*mdx*=71.8%, dKO=79.2%, Fig. 3c). We also examined isolated intact single fibres from the EDL of WT, *mdx* and dKO mice for altered branching profiles (Fig. 3d). Fibre branches are known to accumulate over time as a marker of muscle damage in *mdx* mice^30^. Fibres isolated from dKO showed a small reduction in the number branched compared with *mdx* (Fig. 3e), but we observed a drastic reduction in the complexity of branching in dKO as only 18% of fibres contained four or more branches, compared with 74% of *mdx* (Fig. 3f).

### *ACTN3* genotype modifies disease progression in DMD patients

**Figure 4:**
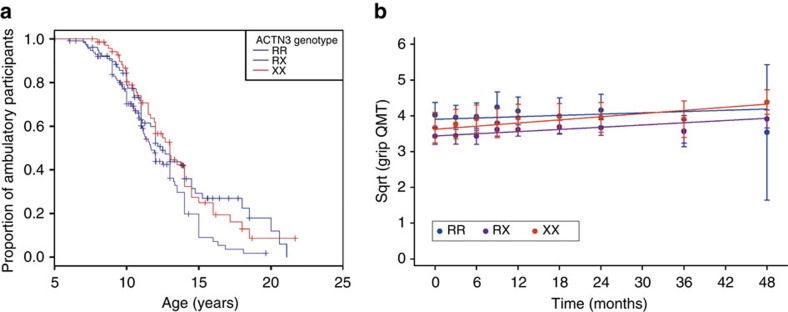
Longitudinal analysis of the CINRG cohort. (**a**) Kaplan-Meier survival analysis for age at loss of ambulation (LoA) in 266 CINRG Duchenne Natural history Study participants. Heterozygous (RX) participants suffered 1- to 2-year earlier LoA with a median age of 11.7 years (95% CI 11.0–13.0), as opposed to 12.5 (11.1–14.0) in RR and 13.0 (11.9–14.0) in XX. (**b**) Longitudinal analysis of grip QMT in steroid-treated 6 to 10 year old DMD patients. They are not necessarily the same subjects throughout the 48 month period of testing. Some subjects only met the criteria in the middle of the study and others were only included in the analysis for a few time points. Error bars represent mean±95% CI.

Given that α-actinin-3 deficiency ameliorates disease progression in *mdx* mice, we tested the effect of *ACTN3* deficiency against the progression of DMD in the CINRG DNHS cohort by analysing LoA and repeated measures of grip QMT over time. Genotype-specific Kaplan-Meier plots for LoA in 266 CINRG Duchenne Natural History Study participants show that heterozygote (RX) participants suffered 1-year to 2-year earlier LoA (Fig. 4a): median age at LoA in these participants was 11.7 years (95% CI 11.0–13.0), but no difference was observed between RR (12.5 years, 95% CI 11.1–14.0) and XX (13.0 years, 95% CI 11.9–14.0). The same pattern was detected when restricting the analysis to European ancestry: median age at LoA in the RX genotype was 12.0 years (95% CI 11.0–14.0), versus 13.0 (95% CI 11.1–18.0) in RR and 14.0 (95% CI 12.0–14.5) in XX. Taking the RR genotype as reference and performing a Cox regression, including glucocorticoid (GC) treatment as a covariate, the RX genotype was significantly associated with risk of earlier LoA, with an odds ratio (OR) of 1.44 (95% CI 1.01–2.07, *P*=0.044). In the smaller European ancestry sub-cohort, there was a trend in the same direction using the same statistical test: OR for the RX genotype was 1.64 (95% CI 0.93–2.90, *P*=0.089). We genotyped the Padova DMD cohort for rs1815739 (*n*=102 successfully genotyped), and again observed an earlier median LoA in RX (10.4 years, 95% CI 9.92–11.6) than RR (11.0 years, 95% CI 10.32–12.7, Supplementary Fig. 2), but this difference was not significant in a Cox regression model including GC treatment. Median age at LoA in the XX group was 10.2 years, 95% CI 9.8 – undetermined upper limit because of a low number of individuals with this genotype (*n*=10). To test concurrent effects of *ACTN3* genotype, previously known genetic modifier genotypes (*SPP1* rs28357094 and *LTBP4* rs10880), and GC treatment, we devised a Cox regression model including 297 participants of self-identified European ancestry, from both the CINRG DNHS and Padova Cohort, for whom all these data were available. In this model, the effect of the *ACTN3* RX genotype was detrimental (OR 1.45, 95% CI 1.05–2.00, *P*=0.022), while the homozygous XX genotype was not (Supplementary Table 1). The strong effect of GCs in delaying LoA was confirmed by this analysis, which showed a 3.1 year delay of LoA with treatment (OR 0.23, 95% CI 0.16–0.31, *P*<0.0001, see Supplementary Table 1), stressing the importance of including GC treatment in the statistical model for genotype effects.

We also examined grip QMT over a 4 year period in steroid treated 6–10 year olds using mixed effects linear regression (Fig. 4b). Similar to our findings at baseline, we see a reduction in strength associated with the X allele as RX individuals show a mean grip strength which is 0.471 lbs less than RR (95% CI 0.172–0.770, *P*=0.002) and 0.283 lbs less for XX individuals (95% CI 0.062–0.628, *P*=0.108). When we combined all genotypes, we did not see a difference in grip strength over time, suggesting that the dystrophic pathology counteracts the natural increase in strength one would expect during childhood development between these two ages. However, we did see a significant interaction between *ACTN3* genotype and time (*P*=0.021). This suggests that *ACTN3* genotype significantly influences grip strength over time, although the magnitude of this influences was not evaluated at the individual genotype level, but instead as a single genotype factor.

### Increased calcineurin activity ameliorates pathology in dKO

Slow fibres are largely spared in the pathogenesis of DMD (ref. 24), and forced fast-to-slow fibre type conversion via the overexpression of calcineurin ameliorates the dystrophic phenotype in *mdx* mice^25^,^26^. The exact mechanism by which slow fibres are resistant to dystrophy is unclear, but may be the result of one or more of the following properties - smaller fibre diameter, increased utrophin expression or a preference for oxidative metabolism. We sought to test each of these possible explanations for the protective effect of α-actinin-3 deficiency on disease progression in *mdx* mice.

**Figure 5:**
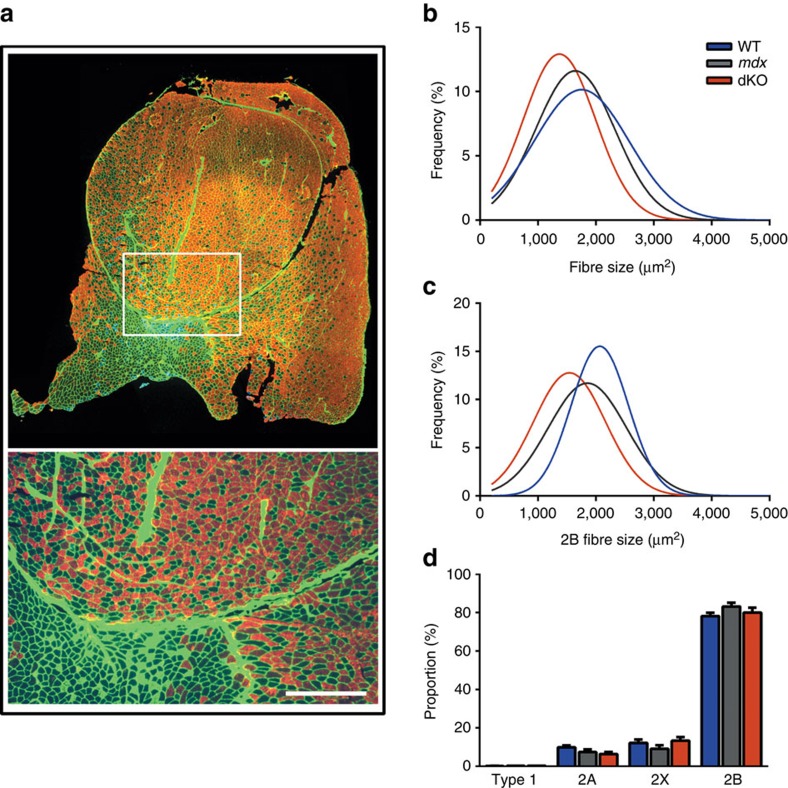
Fibre size is reduced in dKO mice. (**a**) Representative image of fibre typing in WT quadriceps. Sections cut from the midbelly of 12 month old male quadriceps were stained with antibodies against MyHC Type 1 (green), 2A (blue) and 2B (red), while 2X fibres were left unstained. A488 conjugated wheat germ agglutinin was used to mark membranes to delineate the individual fibres (Green). Scale bar indicates 500 μm. (**b**) Fibre size frequency distribution for all quadriceps fibres (Approx 7,000 per quadriceps). The mean fibre size was reduced in dKO compared with *mdx* (1781±158 μm^2^ versus 1223±242 μm^2^, *P*<0.05). (**c**) Frequency distribution for all 2B fibres in the quadriceps. Mean 2B fibre size is reduced in dKO compared with WT (1950±213 μm^2^ versus 1670±267 μm^2^, *P*<0.05). (**d**) Individual fibre types presented as a percentage of total fibre number show no shift in fibre type proportion across all the genotype groups. Data shown as mean±s.e.m., WT *n*=6, *mdx n*=7, dKO *n*=6.

We cut sections from the mid-belly of the quadriceps and co-labelled with antibodies against the different myosin heavy chain (MyHC) isoforms to investigate fibre size and type distribution (Fig. 5a). At 12 months, both *mdx* and dKO showed a higher frequency of small fibres compared with WT (Fig. 5b). While this can be attributed in part to the presence of small regenerating fibres in dystrophic muscle, fibre branches are also likely included in this analysis as these are difficult to identify in cross-section and hence are included in the counts. We observed a significant reduction in overall fibre size in dKO muscle compared with WT (Fig. 5b), which is likely a product of reduced 2B fibre size in these animals (Fig. 5c). However, we did not observe a shift in fibre type as the relative proportions in MyHC isoform were unchanged (Fig. 5d).

**Figure 6:**
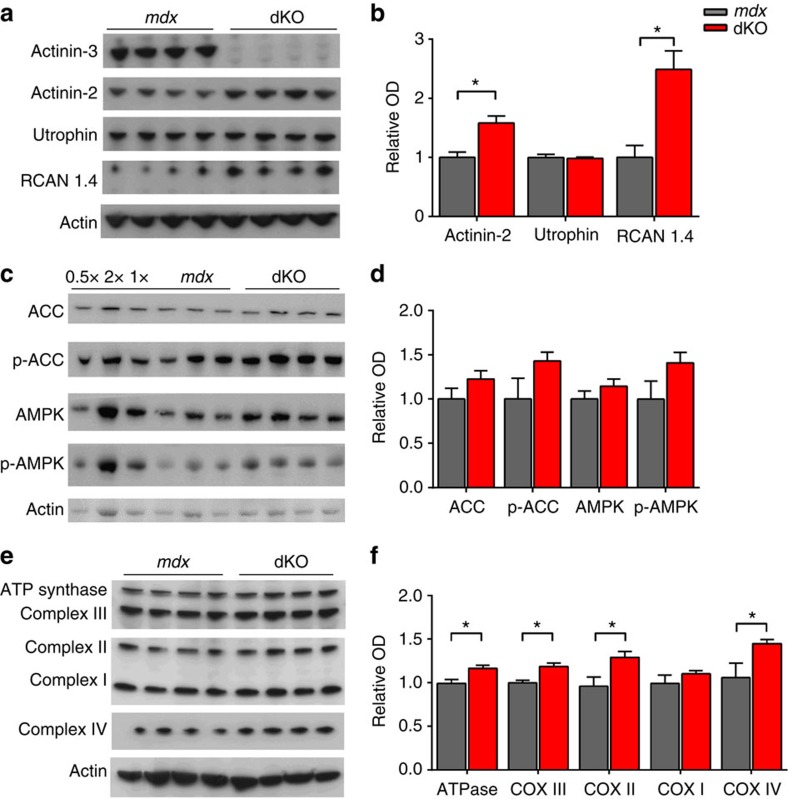
Relative protein expression in dKO muscle compared to *mdx*. (**a**,**b**) dKO muscle at 12-months of age show a compensatory upregulation of α-actinin-2 for the loss of α-actinin-3 and a ∼2.4-fold upregulation in RCAN1.4, but no change in the level of utrophin. (**c**,**d**) No significant differences in the expression of total and phosphorylated ACC, and total and phosphorylated AMPK were observed, but dKO mice did show a trend for increased expression of each. (**e**,**f**) dKO muscle shows an increase in oxidative metabolism as evidenced by significant upregulation in ATP synthase (ATPase), succinate-Q oxidoreductase (Complex II), cytochrome-c oxidoreductase (Complex III) and cytochrome c oxidase (Complex IV). Graphs **b**,**d** and **f** represent relative OD levels normalized to actin and mdx. Data shown as mean±SEM, One-way ANOVA, *P< 0.05. *mdx n*=4, dKO *n*=4. Full-length blots are presented in Supplementary Fig. 6.

At a protein level, there is a significant increase in the level of α-actinin-2 in dKO (Fig. 6a,b); this is a characteristic of α-actinin-3 deficient muscle in *Actn3* KO mice, and likely represents a compensatory upregulation of this closely related protein^21^,^31^. In addition, we have previously observed a significant increase in calcineurin activity in both α-actinin-3 deficient human and mouse muscle, and demonstrated that this is a likely mechanism for the slower twitch relaxation time, increased recovery from fatigue, enhanced response to endurance training and shift towards an oxidative metabolic phenotype in *Actn3* KO mice^20^. Calcineurin is a calcium sensitive phosphatase known to signal fast-to-slow fibre type transition in skeletal muscle^32^. Here we show a marked (2-fold) upregulation in the expression of regulator of calcineurin 1.4 (RCAN 1.4) (which is indicative of increased calcineurin signalling) in dKO mice compared with control *mdx* (Fig. 6a,b); however this was not associated with an increase in the expression of utrophin or a shift in fibre type (based on MyHC isoform) at baseline (Supplementary Fig. 3).

AMP-activated protein kinase (AMPK) is another key regulator of muscle metabolism and also plays a role in the conversion of fast muscle fibres to a slow fibre phenotype^33^. Pharmacological stimulation of AMPK in *mdx* mice causes a shift towards oxidative metabolism and ameliorates the dystrophic pathology^34^,^35^. On this basis, we examined the total and phosphorylated expression levels of AMPK and the co-regulated protein acetyl-CoA carboxylase (ACC) in *mdx* and dKO muscle and observed trend for increased AMPK and ACC phosphorylation expression at baseline (Fig. 6c,d), providing further novel evidence for a shift towards a more oxidative muscle metabolism in α-actinin-3 deficient *mdx* muscle.

Given the increase in two of the key regulators of oxidative metabolism (calcineurin and AMPK) we assessed the dKO mouse at baseline for changes in *Pgc-1α* mRNA expression and markers of oxidative phosphorylation. In dKO muscle we observed a ∼2-fold increase in *Pgc1-α* mRNA when normalizing mRNA expression to the geometric mean of four reference genes (One-way ANOVA, *P*=<0.01), with no obvious change in protein expression (Supplementary Fig. 4). We also see evidence for a shift in mitochondrial function as dKO muscle shows upregulation in the expression of ATP synthase (∼16%, ATPase), succinate-Q oxidoreductase (∼30%, Complex II), cytochrome-c oxidoreductase (∼18%, Complex III) and cytochrome c oxidase (∼44%, Complex IV) compared with *mdx* controls (Fig. 6e,f).

## Discussion

Recent advances in muscular dystrophy research have resulted in an increased number of potential therapies and subsequent clinical trials to treat DMD and slow disease progression^36^,^37^. While this is exciting, the detection of unequivocal therapeutic benefit in these trials has been challenging and they are not currently approved for use by patients. One reason for this lack of translation into clinical practice is because of the absence of clear positive outcomes from current clinical trial data^38^. Though this may be because of failed utility of the drug or treatment, the large inter-patient variability in response to many of these agents has made it difficult to draw conclusions (positive or negative) from many of these studies. It is increasingly recognized that the identification of genetic modifiers is vital to minimize the confounding effects of inter-patient variability in these trials. Here we present evidence that a common null polymorphism in the *ACTN3* gene has a significant effect on muscle strength and function in young ambulant DMD patients. The *ACTN3* 577X allele was associated with significantly reduced muscle strength and poorer performance in the 10 m walk test. Interestingly, the *ACTN3* 577XX genotype is also associated with reduced 40 m sprint times in healthy adolescent males^15^, which mirrors our findings in DMD patients. Muscles from dKO mice also produce significantly less force than their *mdx* counterparts, but show increased recovery from fatigue. Taken together, these data strongly suggest that α-actinin-3 deficiency results in reduced power output in dystrophic muscle and that *ACTN3* R577X genotype should be considered when evaluating quantitative muscle tests in DMD patients.

Susceptibility to stretch-induced damage is a well characterized phenotype in dystrophic muscle^28^,^39^,^40^, and it worsens as the disease progresses^30^. The young (8-week old) dKO mice show a greater percentage of EBD containing muscle fibres following a single downhill run. However, there was no difference in susceptibility to eccentric contraction damage when measuring isolated *ex vivo* EDL of young dKO compared with *mdx* control mice. This is an important comparison. In isolation the downhill run analysis would suggest that the absence of α-actinin-3 results in a larger number of damaged muscle fibres; however *ex vivo* analysis of EDL muscles suggest that there is no effect of genotype in muscle damage properties at 8 weeks of age. In this case, the *in vivo* approach of downhill running is likely confounded by the observation that *Actn3* KO mice are able to run further and more effectively than WT mice^20^. The isolated EDL analysis removes the intrinsic aspect of *Actn3* genotype-associated running performance from this analysis and allows us to observe the primary effect of α-actinin-3 deficiency. Taking both data into account, it is reasonable to assess that dKO muscle shows a similar susceptibility to stretch-induced damage compared with *mdx* at 8 weeks.

At 12 months old however, α-actinin-3 deficiency exhibits a significant protective effect against stretch-induced damage, with dKO mice showing a ∼34% reduction in force deficit following eccentric contraction. Given that we saw no difference between dKO and *mdx* at 8 weeks, and that susceptibility to stretch-induced damage is known to increase with age, this suggests that α-actinin-3 deficiency alters the *mdx* pathology over time. Because this is an important finding, we re-tested the phenotype in the TA using an *in situ* muscle function assessment which some consider to be the gold-standard for examining functional differences in muscle performance in rodents. Again we saw a significant amelioration in the susceptibility to stretch-induced damage in dKO muscle compared with *mdx*, confirming our findings from the EDL. Finally we looked at fibre branching morphology, as branched fibres are also known to accumulate with disease progression in dystrophic muscle^41^ and correlates with susceptibility to stretch-induced damage^42^. The observed reduction in branching complexity in dKO mouse muscle at 12 months directly correlates with the decreased susceptibility to eccentric damage. In combination, these data provide strong evidence to suggest that dystrophic progression is slowed in α-actinin-3 deficient muscle.

While we show evidence that α-actinin-3 deficiency ameliorates disease progression in *mdx* mice, examining an effect of *ACTN3* on progression in DMD patients is much more challenging. *ACTN3* genotype has a significant effect on LoA in the CINRG cohort, but this is largely driven by a reduced ambulation in heterozygous (RX) patients compared with both homozygous groups. Biologically, this is difficult to explain, but we have recently shown evidence for haploinsufficiency associated with *ACTN3* (ref. 43), which may explain this phenomenon. Preliminary analysis shows that *Actn3*^*+/−*^ *mdx* muscles display reduced force (comparable to dKO), but not the protection against stretch-induced damage seen in dKO muscle (Supplementary Fig. 5). Although further investigation is required, it appears that loss of a single *ACTN3* allele does not drive muscle adaptation enough to ameliorate the progression of the dystrophic phenotype in *mdx* mice and hence heterozygous mice presumably suffer the disadvantage associated with reduced α-actinin-3 expression (that is, reduced strength), but do not gain any of the associated advantageous phenotypes (protection of muscle damage).

In human populations, *ACTN3* association studies may also be confounded by population stratification, as the null allele is more frequent in non-European populations (1000 Genomes), who might also present a different DMD phenotype severity because of standard of care disparity or different genetic background. Age at LoA in the heterozygous genotype was earlier in both the CINRG European ancestry sub-cohort, and the Padova cohort (also of European ancestry). In the latter, the XX genotype behaved more similarly to RX (early LoA) rather than to RR as in the CINRG cohort, but the number of XX individuals was low (*n*=10), making this estimate scarcely accurate. In the merged European populations (*n*=297), the association of earlier LoA with the heterozygous genotype proved significant independent of the effects of other known genetic modifiers and corticosteroid treatment. When the LoA analysis was restricted to European ethnicity, XX individuals showed prolonged ambulation of 1 year on average compared with RR, but this difference was not significant. The observation of a detrimental effect of the heterozygous, but not of the null homozygous genotype, was unexpected. LoA is a more complex phenotype than sheer muscle strength, as ambulatory function involves compensatory mechanisms, relative involvement of different muscle groups, and joint contractures. Thus, it could be that the metabolic and fibre type changes induced by *ACTN3* deficiency at various levels affect the balance between these variables in a complex manner. Further genotyping of larger cohorts is warranted to strengthen this conclusion.

We made a similar observation when analysing grip strength over time, as *ACTN3* genotype had a significant effect, but we lacked the statistical power to compare individual genotypes. However, scrutinizing the results presented in Fig. 4b suggests that XX individuals show a favourable gradient relative to RR, indicating the α-actinin-3 deficiency may protect against the loss of strength over time. It is clear from both the LoA and longitudinal grip strength analyses that any effect of *ACTN3* on the progression of DMD is subtle and hence large scale cohort analysis will be required to determine the effect with any certainty. However, we do present strong evidence indicating that *ACTN3* genotype modifies muscle strength in DMD and therefore should be included as a covariate in the analysis of functional outcomes for all future clinical trials.

Slow fibres are relatively spared from degeneration in both DMD biopsies^24^ and *mdx* mice^28^; understanding the basis of this ‘protective effect' of slow fibres could provide important insights into potential therapies for DMD. Promoting fast-to-slow fibre type conversion by calcineurin overexpression^25^,^26^ or chronic pharmacological activation of AMPK (refs 34, 35) in *mdx* mice ameliorates the dystrophic pathology and protects against stretch-induced damage. Both interventions result in a decrease in fibre size, increased utrophin expression and a shift towards oxidative metabolism; each of these characteristic properties of slow fibres may modify the dystrophic phenotype. dKO muscles show a reduction in overall fibre size, which is a product of smaller 2B fibres as these are the major constituent of the quadriceps and the only fibre type which expresses α-actinin-3 (ref. 44). However, we did not see a change in the expression of utrophin, but this is consistent with the lack of fast-to-slow fibre type conversion in dKO muscle. Interestingly, dKO muscle displays a physiological increase in calcineurin (RCAN1.4) and AMPK signalling, which result in a similar phenotype to the aforementioned models, including; reduced force production, increased recovery from fatigue and protection against stretch-induced damage. In line with these studies, we see a shift in the contractile properties of dKO muscle towards a slower twitch phenotype, which is accompanied by increases in markers of oxidative metabolism as demonstrated by increased *Pgc1-α* mRNA expression (Supplementary Fig. 4) and upregulated ATP synthase, complex II, III and IV protein expression.

We have previously demonstrated that α-actinin-3 deficiency results in a shift toward a slow-twitch, oxidative metabolic phenotype^18^,^21^, which is known to be impaired in *mdx* muscle^45^,^46^ and contributes to pathology by compromising skeletal muscle integrity^47^. Hence, it is likely that the shift towards oxidative metabolism associated with α-actinin-3 deficiency underlies the protective effect observed in dKO mice. Previous studies have shown that manipulation of either calcineurin^25^,^31^ or AMPK signalling^35^,^36^ ameliorates the pathology of *mdx* mice. But, in both cases, the approaches used increased activity several fold above wild type levels; here we show that physiological alterations in these pathways by a single genetic variant is enough to have a significant effect. This has important implications for the management of DMD patients moving forward; many current treatment strategies including mysotatin inhibition aim to ameliorate progression by inducing muscle hypertrophy to counteract muscle weakness^48^, but our results from α-actinin-3 deficient muscle suggest that pharmacological and exercise interventions which promote a shift towards slow, oxidative metabolism and reduced fibre size may be more beneficial in the long term.

Metabolic dysfunction is a common feature across many different genetic muscle diseases^7^,^49^ and the age-related decline in muscle function^50^,^51^. Hence, we believe that the genetic modifier effect of *ACTN3* is not limited to DMD, but is likely to be more broadly applicable to a wide cross-section of muscle pathologies.

## Methods

### Statistical analysis of the CINRG and Padova human cohorts

We received approval from ethical standards committees on human experimentation (institutional or regional) for any experiments using human subjects, and written informed consent was obtained from all patients (or guardians of patients) participating in the study (consent for research), as previously reported for the CINRG (refs 5, 52) and Padova^5^ cohorts. Velocity and QMT values from the CINRG natural history cohort were tested for normality using the Shapiro–Wilk normality test and visual inspection of histograms. All QMT values were found to be skewed; therefore all analyses were performed on square root transformed QMT values. Mean values were compared among *ACTN3* genotypes via one-way analysis of variance with post-hoc pair-wise comparisons done and adjustment of *P*-values for multiple comparisons using the Šidák method. *P* values≤0.05 were considered statistically significant. Muscle biopsies from this cohort were not available for further molecular analysis.

Further analysis of velocity and QMT values over time were performed using mixed effect linear regression models. The models were fit with velocity or QMT as the dependent variable, genotype and time as fixed effects and subject with a random intercept and slope.

Loss of independent ambulation (LoA) in the CINRG Duchenne Natural History Study (DNHS) was defined as continuous wheelchair use, confirmed by inability to walk 10 m unassisted. Genotyping was performed by a TaqMan genotyping assay. The effect of *ACTN3* genotype on LoA was studied in a time-to-event statistical framework, with age as a time variable and LoA as event, and censoring participants who were still ambulatory at last follow-up. Genotype effect on LoA was tested by Cox regression, with glucocorticoid (GC) treatment as a covariate (treatment for at least 1 year while ambulatory versus untreated or treated <1 year), and modelling genotype as a 3-way categorical variable (RR, RX or XX), as an additive effect (number of copies of the X allele), or as a dominant effect (no copies versus at least 1 copy of the X allele). This statistical analysis was run in R version 3.2.1 (‘survival' package). Minor allele frequency for rs1815739 varies between ethnicities, and as there are differences in phenotype between different races and ethnicities in the CINRG-DNHS cohort, this could lead to a population stratification bias. Therefore, analyses were run both in the whole (multi-ethnic) CINRG-DNHS cohort (*n*=266), and in a sub-cohort of European and European American ancestry, controlled for population stratification by multidimensional scaling analysis (*n*=114)^52^. We also aimed to replicate the results from the CINRG-DNHS in a second cohort of DMD patients. To investigate this, the same methodological approach described above was used to analyse the effect of *ACTN3* genotype on LoA in the Padova cohort of DMD patients (*n*=102).

### Animals

*Mdx* (C57BL/10ScSn-*Dmd*^mdx^/J) mice were obtained from Jackson Laboratories (Bar Harbor, ME, USA). The *Actn3* knockout (KO) line created in this laboratory^21^ was backcrossed onto the C57BL/10 background for 10 generations before being crossed with the *mdx* line to produce mice lacking both α-actinin-3 and dystrophin (dKO). All animal experiments were approved by the Childrens Medical Research Institute (CMRI) and Murdoch Childrens Research Institute (MCRI) animal ethics committees and performed according to their prescribed guidelines. Experiments were performed on male animals at 2 timepoints; 8±1 weeks (baseline) and 12±1 months (aged).

### Muscle physiology assessment

Downhill run was performed in order to induce muscle damage *in vivo* using a modified protocol^53^. Briefly mice were forced to run downhill in the presence of Evans blue dye (EBD) injected intraperitoneally 30 min before performing the downhill run protocol. Following EBD injection mice were placed on an Accupacer treadmill (Columbus Instruments) set at a 15° decline and run for 10 min at a speed of 15 m per min. Mice were euthanized 24 h after the downhill run, and all hindlimb muscles were harvested. To visualize muscle damage, 8 μm sections were cut from the midpoint of the gastrocnemius muscle. These were fixed with 4% paraformaldehyde at 27° for 10 min and then incubated with Alexa488-conjugated wheat germ agglutinin (W11261, Life Technologies, 1:200) for 1 h to mark the boundaries between fibres. EBD inclusion was imaged using fluorescent microscopy, at 620 nm (excitation) and 680 nm (emission). All Fibres were then counted to determine the % of dye inclusion per cross section.

*Ex vivo* Extensor digitorum lognus (EDL) muscle physiology was performed using WT, *mdx* and dKO mice. The EDL was carefully dissected from the lower hindlimb and tied by its tendons to a force transducer (Fort series, World Precision Instruments) at one end, and a linear tissue puller (University of New South Wales) at the other using silk suture (Deknatel 6.0). The isolated muscle was placed in a bath continuously perfused with Krebs solution, composed of (in mM) 4.75 KCl, 118 NaCl, 1.18 KH_2_PO_4_, 1.18 MgSO_4_, 24.8 NaHCO_3_, 2.5 CaCl_2_ and 10 glucose with 0.1% fetal calf serum and was continuously bubbled with carbogen (95%O_2_, 5%CO_2_) to maintain pH at 7.4.

The muscle was stimulated by delivering a supramaximal current between two parallel electrodes in the bath using an electrical pulse generator (A-M Systems). The optimal length (*L*_O_) was determined as the muscle length at which maximum twitch force was generated prior to experimentation and set as the baseline.

A force frequency curve was obtained by delivering 500 ms stimuli of different frequencies (2, 15, 25, 25, 37.5, 50, 75, 100, 125 and 150 Hz) and measuring the force produced during each stimulation. A 30 sec rest was allowed between each to mitigate the effects of fatigue. A curve relating to the muscle force and stimulation frequency was fitted to the data. From the fitted curve the following parameters were obtained; maximum force (*P*_max_), Hill coefficient and twitch to tetanus ratio.

Following generation of the force frequency curve, the muscle was subjected to a series of eccentric stretch contractions. Two distinct protocols were performed to ensure adequate muscle damage was achieved at each time point. We have previously shown that a 15% stretch in young (6-8 week old) *mdx* mice is insufficient to induce muscle damage^30^. Therefore at 8 weeks of age, muscles were stimulated for 4 sec at 100 Hz, and at *t*=250 ms when the maximum isometric force was reached, and stretched to 20% longer than *L*_O_ at a constant speed of 5 mm per sec. The muscle was held at this extended length for 2 sec before being returned to *L*_O_ at 5 mm per sec. This process was repeated six times, with a 3 min interval between each eccentric contraction. Following the final stretch, the muscle was allowed to recover for 10 min before a second force frequency curve was generated. The *P*_max_ following the eccentric contractions was obtained from this and subtracted from the original *P*_max_ to give the raw force deficit. Finally, the raw force deficit was expressed as a percentage of the pre-stretch *P*_max_ for analysis. This protocol was altered for muscles from aged *mdx* mice because of the more advanced disease; muscles were stimulated for 5 sec at 100 Hz, and stretched to 15% longer than *L*_O_ at a constant speed of 2 mm per sec. The muscle was held at this extended length for 2 sec before being returned to *L*_O_ at 2 mm per sec. This process was repeated three times at 5 min intervals and the muscle was allowed to recover for 10 min before the force deficit was calculated.

A separate set of muscles were used to analyse the recovery from fatigue. Following generation of a force frequency curve and after a 10 min rest, muscles were subjected to the following fatigue protocol; repeated 1 sec tetani at 100 Hz every 2 sec for a period of 30 sec. To track the recovery, the force produced by short (250 ms) 100 Hz tetani was recorded at 30 sec, 1, 2, 3, 5 and 10 min post fatigue. The force produced during each of the 100 Hz tetani during the fatigue protocol and subsequent recovery was recorded and expressed relative to the pre-fatigue force.

*In situ* assessment of the tibialis anterior (TA) was performed in aged (12 month old) WT, *mdx* and dKO mice (*n*=5–7 mice per genotype) using the Arora Scientific 1300A whole mouse test system and 701C stimulator. Briefly, mice were anaesthetized using isoflurane inhalation (0.6 ml per min) and placed on a 37^o^C heated platform, the distal tendon of the TA was surgically isolated and the knee joint exposed. Surgical silk (Silicon coated suture silk 5/0, Covidien) was used to secure the TA tendon to the force transducer (Model 809B *in situ* test apparatus), the foot and knee were clamped in place for stability. Maximum tetanic force (mN), specific force (mN per mm^2^) and the degree of contraction-induced damage (% force loss) were determined, as previously outlined in Percival *et al*.^54^ with minor modifications. Briefly, the TA muscle was adjusted to an optimum length (Lo) to produce maximum twitch force (Pt), and the fibre length (Lf) was calculated using the correction value of 0.6 (Lf=Lo × 0.6). The TA was then stimulated every 2 min at increasing frequencies (5–250 Hz) to generate force–frequency curves. Maximal tetanic force (Po) was typically achieved at 150 Hz. Both Lo and TA mass were recorded and used to determine Pt, Po and specific tetanic (sPo) force for each muscle.

The TA muscle from each subject underwent a series of lengthening contractions of progressively increased strain (0–35%). Strain is the percentage increase in length beyond the optimal Lf. Muscles were maximally stimulated (7 mA, at 150 Hz) for 150 ms at Lo to achieve maximal isometric tension, immediately followed by 200 ms of stimulation during the application of a 5% increase in length (ranging from 0 to 35%) beyond Lf. Stretch was applied at a rate of 2 fibre lengths per sec with 30 sec intervals between each stretch. The force (Po) generated immediately prior to the initiation of the subsequent lengthening contraction was recorded and normalized to the starting force to calculate % force generated.

### Tissue collection

Mice were euthanized via cervical dislocation. The skin was removed from the hindlimb and individual muscles were dissected out, with overlying fascia and adipose tissue discarded. The wet weight of the isolated muscles was recorded immediately upon excision to minimize the effect of evaporation on the measurement. Following this, the individual muscles were covered in a cryo-preservation medium (TissueTek) and frozen in liquid nitrogen-cooled isopentane. Tissue was stored at −80° until use.

### Muscle histopathohlogy and fibre morphology

Histological assessments were performed in the TA of 12 month old *mdx* and dKO mice (*n*=10–14 mice per genotype). Briefly, the TA of WT, *mdx* and dKO mice were removed and weighed before being frozen in liquid nitrogen cooled isopentane, samples were stored at −80 °C until use. The TA were cut through the mid-belly and 8 μm sections were collected and placed on Superfrost-Plus microscope slides (Thermo fisher Scientific). Hematoxylin and eosin (H&E) staining was performed as outlined previously^55^. Muscles were frozen in liquid nitrogen pre-chilled isopentane and stored at −80 °C until analysis. Muscles were cryo-sectioned at 8 μm and placed on a microscope slide. The sections were incubated in Haematoxylin (for 5 min, HHS32, Sigma Aldrich) and Eosin (for 2 min; HT110232, Sigma Aldrich) and the whole TA cross-section was imaged using a scanning light microscope (Leica Biosystems). Images were imported into ImageJ for analysis^56^. All histological analyses were performed according to the Treat-NMD preclinical standard operating procedures (SOPs) while blinded to genotype.

Fibre branching morphology was performed using individual fibres isolated from the EDL of WT, *mdx* and dKO mice (*n*=3 mice per genotype). The muscles were digested in Krebs solution (in mM): 1.2 NaH_2_Po_4_, 1 MgSO_4_, 4.83 KCl, 137 NaCl, 24 NaHCO_3_, 2 CaCl_2_, 10 Glucose, with 3 mg per ml collagenase IV (C5138, Sigma-Aldrich), which was bubbled with carbogen and incubated at 37 °C. After 30 min, the muscles were removed from the digesting solution, rinsed in Krebs and placed in a relaxing solution (in mM): 117 K^+^, 36 Na^+^, 1Mg^2+,^ 60 HEPES, 8 ATP, 50 EGTA^2−^, with free Ca^2+^ of 10^−7^ M. Individual fibres were obtained by gently agitating the muscle with a pipette and their morphology was examined under a light microscope. Fibre morphological analyses were performed blinded to genotype and digital-photographic representations recorded for all fibres (*mdx*, *n*=272 fibres and dKO, *n*=608 fibres).

### Myosin heavy chain profile

Transverse 8 μm sections were cut from the midpoint of the quadriceps muscle. These sections were blocked with 2% BSA for 10 min before being incubated overnight at 4 °C with an antibody against myosin heavy chain 2B (BF-F3, DSHB, University of Iowa, USA, 1:10 dilution) and Alexa488-conjugated wheat germ agglutinin (W11261, Life Technologies, 1:500). Following primary incubation sections were washed three times with PBS and re-blocked with 2% BSA for 10 min. Secondary antibodies, Alexa555 goat anti-mouse IgM (A-21426, Molecular Probes, 1:500) and Alexa 488 goat anti-rabbit IgG (A-11008, Molecular Probes, 1:500) were then applied for 2 h at room temperature. Sections were washed three times in PBS and blocked with AffiniPure fab fragment goat anti-mouse IgG (115-007-003, Jackson ImmunoResearch, 1:200) for 1 h. Immediately following this, sections were incubated with antibodies against slow myosin type 1 (MAB1628; Chemicon, 1:100) and myosin heavy chain 2A (SC71; DSHB, University of Iowa, used neat), covalently labelled with Alexa 488 and Alexa 350 respectively, with a Zenon labelling kit (Z25070, Invitrogen) for 2 h at room temperature. Finally sections were washed three times in PBS to remove any unbound antibody, fixed with 4% paraformaldehyde for 10 min and coverslips were mounted. These whole IHC-stained sections were imaged at × 40 using ProgRes camera and software (SciTech). The fibre diameter and number was analysed using MetaMorph software according to published protocols^57^.

### Protein expression analysis

The snap frozen quadriceps from each mouse were ground using a liquid nitrogen-cooled mortar and pestle. Approximately 2 mg of the ground tissue was solubilized in 500 μl 4% SDS buffer containing 50 mM Tris, 10% glycerol, 50 mM DTT, bromophenol blue and protease inhibitor cocktail (Sigma-Aldrich). The concentration of total protein in each sample was assessed using a BCA protein assay kit (Thermo Fisher).

Up to 50 μg of protein lysate was loaded onto 4–12% Bis–Tris precast minigels (Life Technologies), separated by SDS–PAGE and transferred onto polyvinylidene fluoride membranes (Millipore). The membranes were incubated with antibodies against α-actinin-2 and -3 (gift from Alan Beggs, Children's Hospital Boston), AMPKα (Total (5,831) and Thr^172^ (2,535) Cell Signalling, 1:1,000), Acetyl-CoA Carboxylase (ACC total (3,676) and Ser^79^ (11,818) Cell Signalling, 1:1,000), utrophin (DRP2-CE-S, Novocastra, 1:1,000), RCAN 1.4 (D6694; Sigma-Aldrich, 1:500), MitoProfile Total OXPHOS antibody cocktail (AB110413, Abcam, 1:2,000), COX IV (20E8; Molecular Probes, 1:1,000) and Pgc1α (AB3242, Merk Millipore, 1:1,000). α-Sarcomeric actin (A2172, 5C5, Sigma-Aldrich, 1:2,000), was used as a loading control, and after developing, membranes were stained with Coomassie Brilliant Blue (1610436, Sigma-Aldrich), and the expression of myosin was used as an additional loading control. Films were scanned using an Epson scanner, and the optical density of bands was computed using ImageJ software (NIH).

### Quantitative real-time PCR (RT-qPCR)

Total RNA was isolated from ∼50 mg of mouse quadriceps tissue using TRIzol (Invitrogen) according to the manufacturer's instructions. The RNA was resuspended into 50 μl DEPC treated water and purified and DNAseI treated using RNeasy Mini on-column clean-up (Qiagen). RNA quality and quantity was assessed using a 2100 Bioanalyser (Agilent Technologies) 6,000 RNA kit (Agilent Technologies) according to the manufacturer's instructions. One microgram (1 μg) of total RNA was reverse transcribed using Super-Script III Reverse Transcriptase (Invitrogen), random pdN6 primers (Roche), 0.1 mM DTT (Invitrogen) and 10 mM dNTP (Invitrogen) in a 20 μl volume for 90 min at 50 °C on a Veriti 96-well thermocycler (Applied Biosystems). All cDNA was diluted in DEPC treated water (Bioline) prior to RT-qPCR. Diluted cDNA was aliquoted into working volumes of 10 μl and stored at −80 °C until use.

Reverse transcriptase quantitative PCR (RT-qPCR) was performed using standard curves generated from serially diluted plasmid constructs of each gene of interest as outlined in Thomas *et al*.^58^ Relative gene expression was determined using the geometric mean of four reference genes (*Rer1*, *Rpl41*, *Rn18S*, *Actb*) to normalize *Pgc1α* mRNA expression in *mdx* and dKO (Table 4). Absolute *Pgc1α* expression levels were determined based on the known DNA concentrations from the *Pgc1α* standard curve using our previously published method^58^. RT-qPCR was carried out using a Rotor-Gene 6000 (Qiagen) in a 20 μl reaction volume consisting of, 1 μl mouse cDNA, 10 μM of forward and reverse primers, × 1 MyTaq buffer (Bioline), 0.2 U MyTaq DNA polymerase (Bioline), 0.4 μl × 10 SYBR Green I (Life Technologies) and DNAse-free water. All samples and plasmid DNA were amplified in triplicate and a no-template-control (NTC) was included in each reaction. Amplification were performed with a 2 min denaturation step at 95 °C, followed by 35 cycles of 95 °C for 30 sec, 62–65 °C (Table 4) for 30 sec and 72 °C for 30 sec. Melt curve analysis was performed from 60–99 °C to assess amplification specificity.

### Statistical analysis of animal data

The methods used for analysis of animal data are the same as those described for the analysis of velocity and QMT phenotypes in the human data, namely analysis of variance methods with *post-hoc* pair-wise comparisons where appropriate. This analysis was performed using GraphPad Prism software.

### Data availability

All relevant data are available from the authors. The human cohort dataset is not publicly available, but can be obtained via formal request to the CINRG consortium (www.cinrgresearch.org).

## Additional information

**How to cite this article:** Hogarth, M. W. *et al*. Evidence for *ACTN3* as a genetic modifier of Duchenne muscular dystrophy. *Nat. Commun.* **8,** 14143 doi: 10.1038/ncomms14143 (2017).

**Publisher's note:** Springer Nature remains neutral with regard to jurisdictional claims in published maps and institutional affiliations.

## Supplementary Material

Supplementary Information Supplementary 1-6 and Supplementary Table 1

## Figures and Tables

**Table 1:** Baseline CINRG natural history cohort analysis.

**Phenotype**	**Overall** ***P*** **value**	***N*: mean±s.d.**	***Post-hoc P*** **values indicating significantly different means**
10 Metre Walk Test (velocity m per sec)	0.0366	RR (*N*=16; 2.22±0.77)*	*P*=0.034*
		RX (*N*=29; 1.69±0.65)*	
		XX (*N*=16; 1.79±0.54)	
Grip QMT†	0.0228	RR (*N*=16; 4.02±0.66)*	*P*=0.019*
		RX (*N*=29; 3.43±0.60)*	
		XX (*N*=16; 3.67±0.79)	
Elbow extensor QMT†	0.0381	RR (*N*=16; 2.84±0.63)*	*P*=0.032*
		RX (*N*=27; 2.36±0.55)*	
		XX (*N*=16; 2.54±0.57)	
Elbow flexor QMT†	0.0641	RR (*N*=16; 3.10±0.56)	NONE
		RX (*N*=27; 2.68±0.60)	
		XX (*N*=16; 2.77±0.52)	
Knee extensor QMT†	0.0222	RR (*N*=16; 4.09±1.15)*	*P*=0.033*
		RX (*N*=27; 3.26±0.95)*	
		XX (*N*=16; 3.24±0.93)	
Knee flexor QMT†	0.0384	RR (*N*=16; 3.72±0.43)*	*P*=0.0384*
		RX (*N*=27; 3.32±0.51)*	
		XX (*N*=16; 3.54±0.54)	

One-way ANOVA, Šidák adjustment for multiple comparisons.*denotes genotype groups compared to give the *P* value shown in column 4.

^†^Square root transformed variable.

**Table 2:** Physiological properties of isolated EDLs from young WT, *mdx* and dKO mice.

**Parameter**	**WT**	***mdx***	**dKO**
EC50 (Hz)	59.4±0.8	55.9±0.3	55.5±1.4
Hill co-efficient	4.2±0.1	4.5±0.1	4.2±0.4
Twitch time to peak (ms)	21.3±2.4	19.3±1.0*	20.2±2.7
Twitch half-relax time (ms)	18.0±472	13.8±2.2*	17.5±4.3^†^

One-way ANOVA.

*P<0.01 versus WT.

†*P*<0.01 versus *mdx.*

**Table 3:** Physiological properties of isolated EDLs from aged WT, *mdx* and dKO mice.

**Parameter**	**WT**	***mdx***	**dKO**
EC50 (Hz)	50.2±0.7	60.9±0.3*	57.0±1.6*
Hill co-efficient	3.8±0.2	4.3±0.2	4.1±0.3
Twitch time to peak (ms)	24.6±0.6	18.0±0.7*	18.9±2.5*
Twitch half-relax time (ms)	17.2±2.4	10.2±0.8*	18.2±6.6^†^

One-way ANOVA.

*P<0.01 versus WT.

†*P*<0.01 versus *mdx.*

**Table 4:** RT-qPCR primers and conditions.

**Gene ID**	**Accession number**	**Forward oligo sequence (5′–3′)**	**Reverse oligo sequence (5′–3′)**	**Product size (bp)**	**Temp (°C)**	**Intron spanning**
*Actb*	NM_007393	CCTCCCTGGAGAAGAGCTATG	TTACGGATGTCAACGTCACAC	157	65	Yes
*Rn18s*	NR_003278	GCAATTATTCCCCATGAACG	GGCCTCACTAAACCATCCAA	124	65	N/A
*Rer1*	NM_026395	GCCTTGGGAATTTACCACCT	CTTCGAATGAAGGGACGAAA	137	62	Yes
*Rpl41*	NM_018860	GCCATGAGAGCGAAGTGG	CTCCTGCAGGCGTCGTAG	113	65	Yes
*Pgc1α*	NM_008904	CAACAATGAGCCTGCGAAC	AGGGCAATCCGTCTTCATC	116	65	Yes

Forward and reverse oligonucleotide sequences (5′–3′), product size and annealing temperature for each reference gene (*Rer1*, *Rpl41*, *Rn18S*, *Actb*) and *Pgc1α*.
