# Supplementary material for: Evidence for ACTN3 as a genetic modifier of Duchenne muscular dystrophy
Source: Nat Commun. 2017 Jan 31;8:14143. doi: 10.1038/ncomms14143 (PMC5290331; doi:10.1038/ncomms14143)
Supplement: Supplementary Information — Supplementary 1-6 and Supplementary Table 1 [file ncomms14143-s1.pdf]

Supplementary Figures:

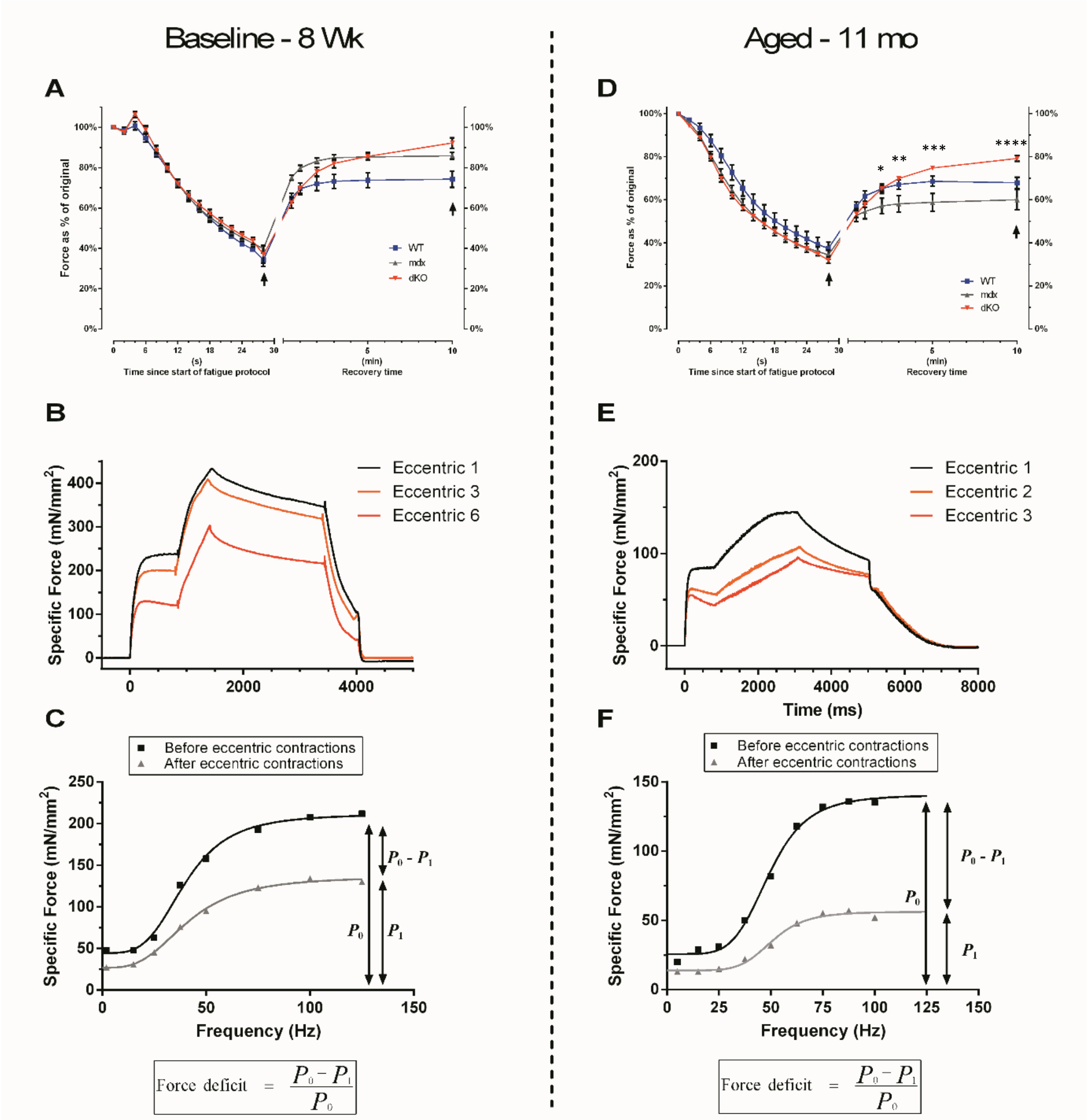

Supplementary Figure 1: Summary of EDL physiology methods from young (A-C) and aged (D-F) mice. A) and **D)** show relative force traces following the fatigue protocol. The timepoints at the end of the fatigue (post-fatigue) and recovery (10 min recover) are indicated by arrowheads. These values were used to generate the data shown in Fig. 1C and Fig 2D. **B)** Shows representative force traces following the 1st, 3rd and 6th eccentric contractions (20% L_O) performed on young EDLs. **C)** Shows the shift in force frequency following the eccentric contraction protocol, and details the calculation of the force deficit displayed in Fig 1D. **E)** Shows representative force traces following the 3 eccentric contractions (15% L_O) performed on aged EDLs. **F)** Shows the shift in force frequency following the eccentric contraction protocol, and details the calculation of the force deficit displayed in Fig 2C. Data shown as mean ± SEM, One-way ANOVA, *p < 0.05, **p<0.01, ***p < 0.001, ****p<0.0001, WT n=5, *mdx* n=6, dKO n=6.

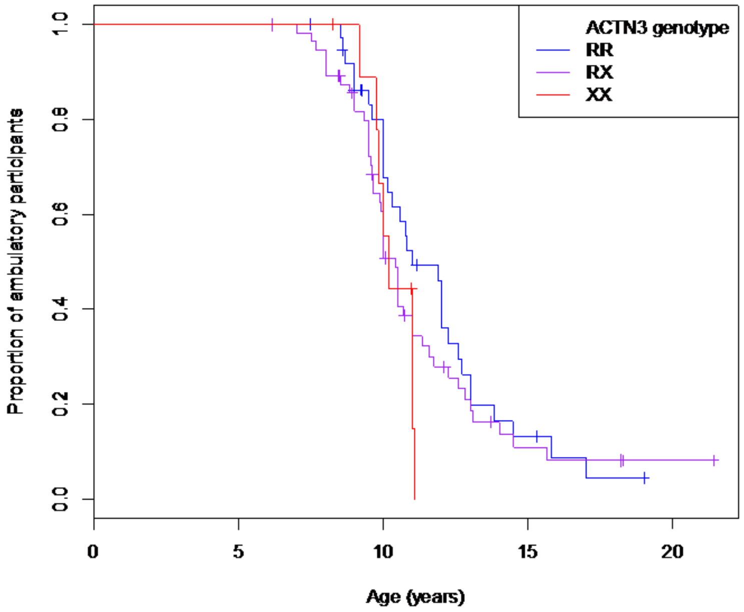

Supplementary Figure 2: Kaplan-Meier plot of the proportion of ambulatory participants by age in the Padova DMD cohort (n = 102).

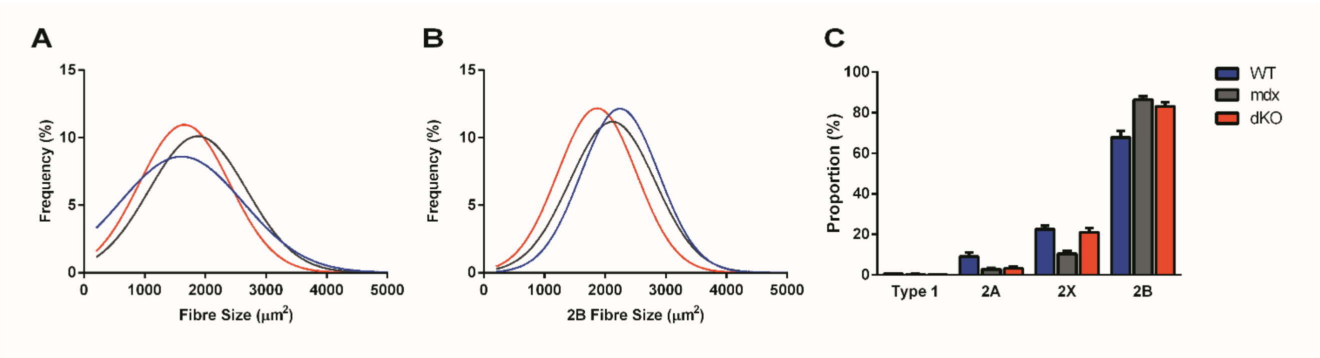

Supplementary Figure 3: Fibre type analysis of young (8wk) quadriceps. A) Frequency distribution of all fibres. **B)** Frequency distribution of 2B fibres. **C)** Relative proportions of each individual fibretype. WT n=6, *mdx* n=8, dKO n=6. Approx. 8000 total fibres counted per animal.

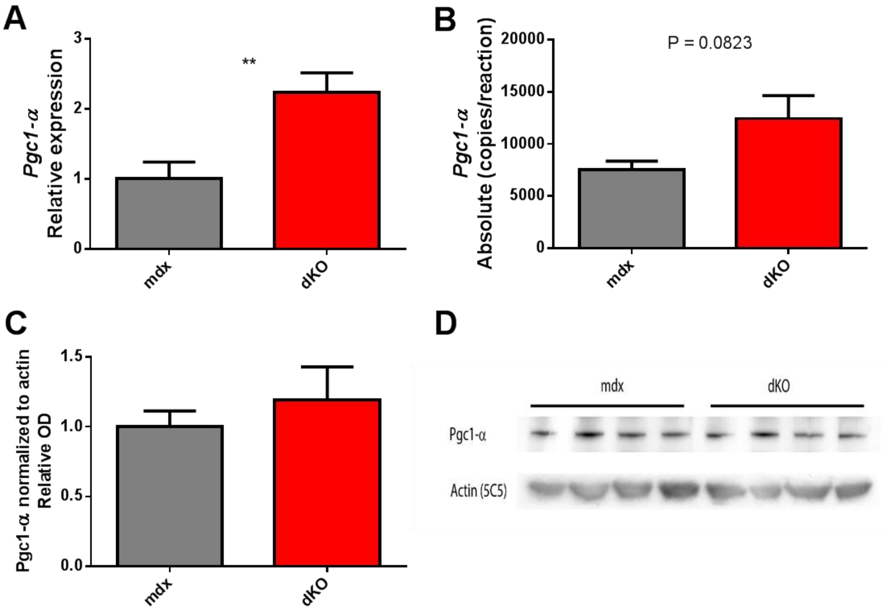

Supplementary Figure 4: Quantitative real-time PCR (RT-qPCR) of *Pgc1-α* **A)** normalised to the geometric mean of four non-significantly different reference genes (*Actb*, *Rer1*, *Rpl41* and *Rn18s*) shows a 2-fold increase in *Pgc1-α* mRNA and **B)** absolute mRNA expression. Data shown as mean, + SEM, n = 6 *mdx;* n = 5 dKO. One-way ANOVA, ** P = <0.01. **C** and **D)** Show no difference in the protein expression of Pgc1-α. Data shown as mean, + SEM, n = 4 *mdx;* n = 4 dKO.

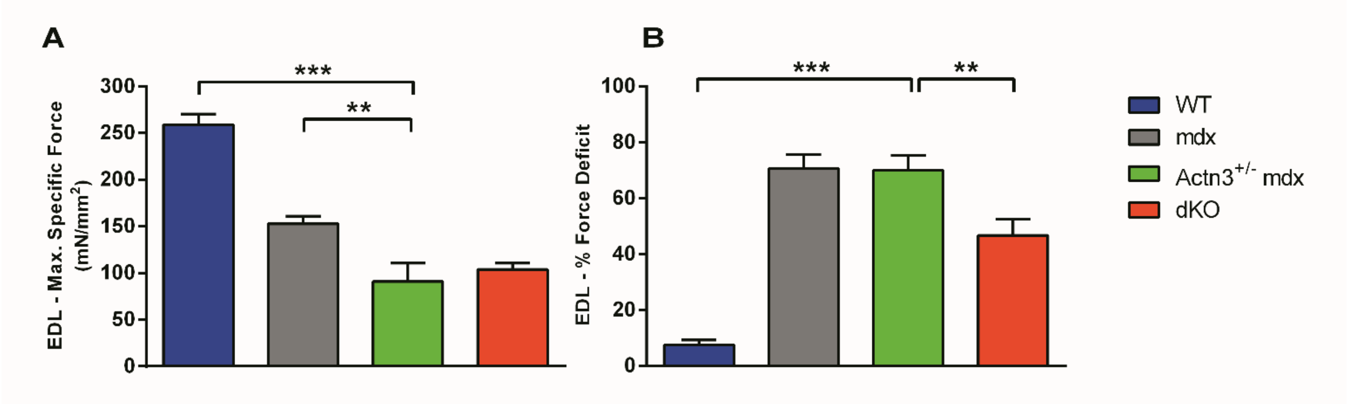

Supplementary Figure 5: Preliminary analysis of EDL muscle physiology from *Actn3$^{+/-}$ mdx* mice. **A)** Shows the data presented in Fig. 2B with the inclusion of *Actn3$^{+/-}$ mdx*. EDLs isolated from *Actn3$^{+/-}$ mdx* produce significantly less force than both WT and mdx (*Actn3$^{+/-}$ mdx*; 90.9 ± 19.8 mN). **B)** Shows the data presented in Fig. 2C with the inclusion of *Actn3$^{+/-}$ mdx* (in green). EDLs isolated from *Actn3$^{+/-}$ mdx* show a significantly larger force deficit following 3 15% eccentric contractions than both WT and dKO (*Actn3$^{+/-}$ mdx*; 70.1 ± 11.8 % loss of force). Data shown as mean ± SEM, One-way ANOVA, **p<0.01, ***p < 0.001. WT n=5, *mdx* n=6, *Actn3$^{+/-}$ mdx* n=5, and dKO n=6.

Supplementary Figure 6: Un-cropped images of western blots presented in the main figures and supplementary figure 4.

Figure 6A:

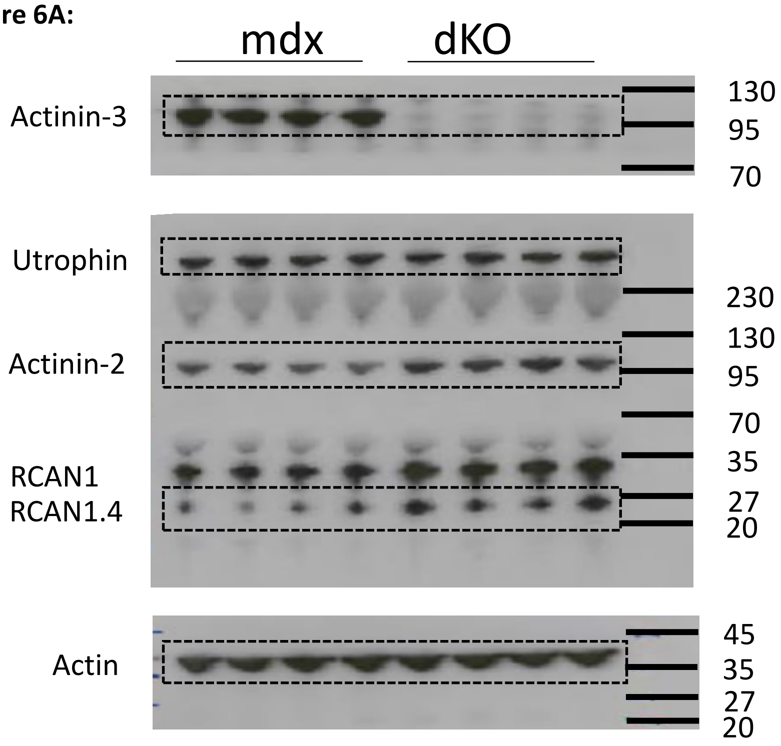

Figure 6B

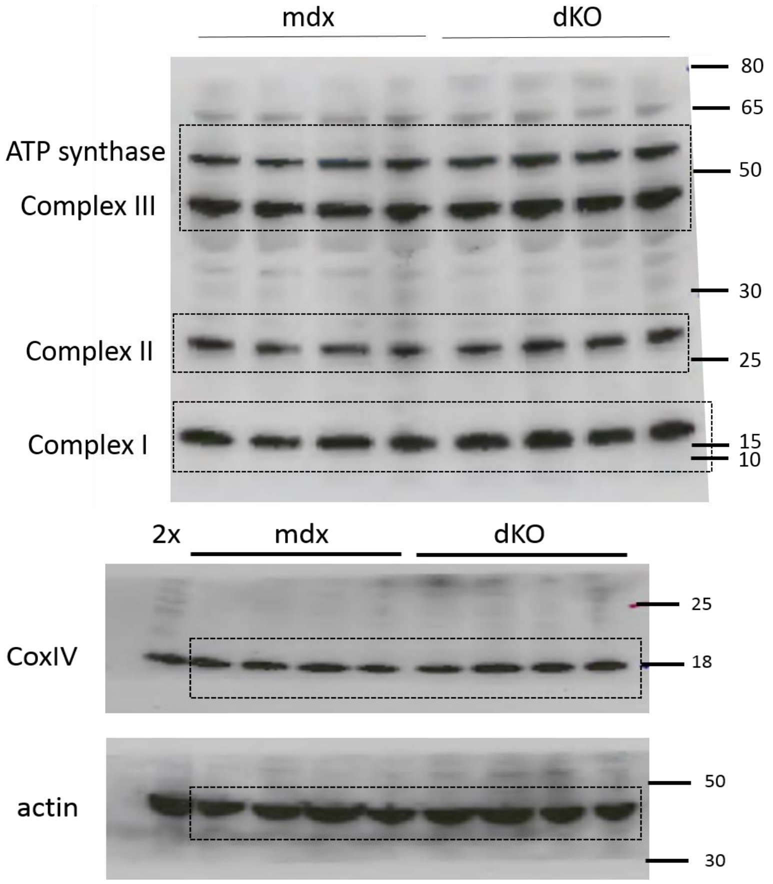

Figure 6C

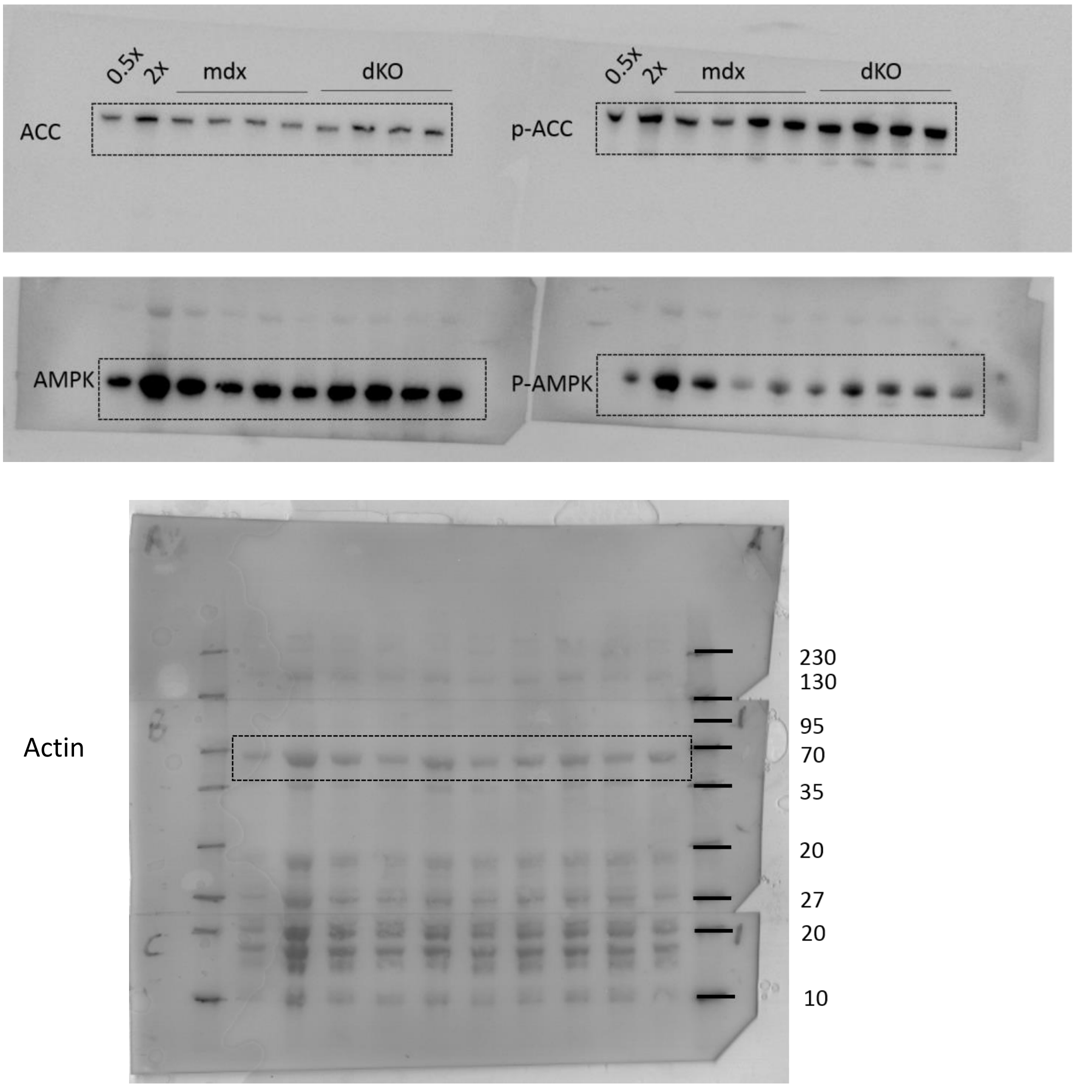

Supplementary figure 4

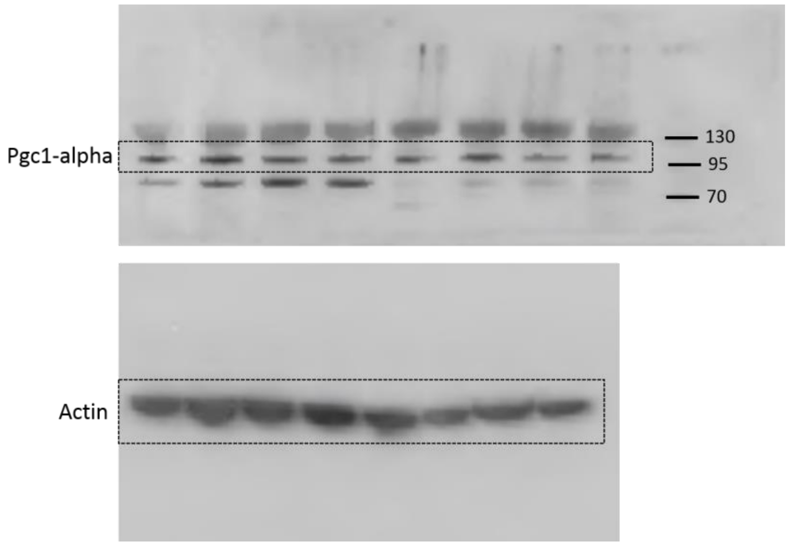

Supplementary Table 1. Survival analysis parameters for loss of ambulation in 297 DMD participants (CINRG and Padova cohorts) of European ancestry.

Covariate	Level of covariate	n	LoA events	Kaplan-Meier analysis		Cox regression analysis		
				median LoA (years)	95% CI	OR	95% CI	p-value
***ACTN3* rs1815739 genotype**	**CC (RR)**	95	67	12	12.0 - 13.0	1*	-	-
	**CT (RX)**	136	93	11.6	11.0 - 12.8	1.45	1.05 - 2.00	***0.022***
	**TT (XX)**	66	42	12.7	11.9 - 14.0	0.96	0.64 - 1.43	0.85
***SPP1* rs28357094 genotype (dominant[5])**	**TT**	203	132	12.7	12.0 - 13.2	1*	-	-
	**TG/GG**	94	70	11.7	11.0 - 12.0	1.32	0.98 - 1.78	*0.067*
***LTBP4* rs10880 genotype (recessive[8])**	**CC/CT**	261	175	12	11.9 - 13.0	1*	-	-
	**TT**	36	27	11.2	10.7 - 14.5	1.31	0.86 - 2.00	0.21
**GC treatment**	**no**	83	73	10	9.5 - 10.5	1*	-	-
	**yes**	214	129	13.1	12.7 - 14.0	0.23	0.16 - 0.31	< ***0.0001***
**Cohort**	**CINRG**	218	140	12.7	12.0 - 13.3	1*	-	-
	**Padova**	79	62	10.7	10.0 - 12.0	1.11	0.79 - 1.54	0.55

DMD: Duchenne muscular dystrophy. **OR:** odds ratio. **SE:** standard error. **CI:** confidence interval. ***ACTN*3:** α-actnin-3. ***SPP1*:** secreted phosphoprotein 1 or osteopontin. ***LTBP4***: latent transforming growth factor beta binding protein 4. **GC:** glucocorticoid corticosteroids. **CINRG-DNHS:** Cooperative International Neuromuscular Research Group Duchenne Natural History Study. * An OR of 1 is set by definition for levels taken as reference in the Cox regression model.
